# Using Green Supplementary Materials to Achieve More Ductile ECC

**DOI:** 10.3390/ma12060858

**Published:** 2019-03-14

**Authors:** Yichao Wang, Zhigang Zhang, Jiangtao Yu, Jianzhuang Xiao, Qingfeng Xu

**Affiliations:** 1 Department of Disaster Mitigation for Structures, College of Civil Engineering, Tongji University, Shanghai 200092, China; wangyichao@tongji.edu.cn; 2 Key Laboratory of New Technology for Construction of Cities in Mountain Area (Chongqing University), Ministry of Education, Chongqing 400045, China; 3 Key Laboratory of Performance Evolution and Control for Engineering Structures of Ministry of Education, Tongji University, 1239 Siping Road, Shanghai 200092, China; 4 State Key Laboratory of Disaster Reduction in Civil Engineering, College of Civil Engineering, Tongji University, Shanghai 200092, China; jzx@tongji.edu.cn; 5 Shanghai Key laboratory of engineering structure safety, Shanghai 200032, China; xuqingfeng73@163.com

**Keywords:** green engineered cementitious composites, recycled powder, crumb rubber, strain hardening, ultra-high ductility

## Abstract

To improve the greenness and deformability of engineered cementitious composites (ECC), recycled powder (RP) from construction and demolition waste with an average size of 45 μm and crumb rubber (CR) of two particle sizes (40CR and 80CR) were used as supplements in the mix. In the present study, fly ash and silica sand used in ECC were replaced by RP (50% and 100% by weight) and CR (13% and 30% by weight), respectively. The tension test and compression test demonstrated that RP and CR incorporation has a positive effect on the deformability of ECC, especially on the tensile strain capacity. The highest tensile strain capacity was up to 12%, which is almost 3 times that of the average ECC. The fiber bridging capacity obtained from a single crack tension test and the matrix fracture toughness obtained from 3-point bending were used to analyze the influence of RP and CR at the meso-scale. It is indicated that the replacement of sand by CR lowers the matrix fracture toughness without decreasing the fiber bridging capacity. Accordingly, an explanation was achieved for the exceeding deformability of ECC incorporated with RP and CR based on the pseudo-strain hardening (PSH) index.

## 1. Introduction

In order to address the brittleness of concrete, two superior materials are developed, that is, ultra-high-performance concrete (UHPC) [1] and engineered cementitious composites (ECC) [2]. UHPC is a kind of concrete which shows high strength and durability, while UHPC has a tension-softening property after peak stress and a low strain-hardening property with the tensile capacity at about 0.5% [3]. López and Serna [4] introduced an innovative design of a 43.5 m span footbridge, constructed only with UHPC and placed over the ovejas ravine in Alicante, Spain. ECC is a special class of high-performance fiber reinforced concrete, which was designed based on the micromechanics theory proposed by Li et al. in the 1990s [2] as a ductile alternative to conventional concrete. Unlike the strain-softening failure mode often observed in the normal fiber-reinforced concrete under tension, ECC exhibits strain-hardening behavior, accompanying the multiple micro-cracks which develop in ECC. The multiple-crack phenomena in ECC, as a result, allow its tensile strain capacity to achieve 3–5%, which is 300–500 times of that of normal concrete [5,6]. Meanwhile, the average crack width in ECC is typically controlled under 100 μm. In previous studies [7,8,9,10], this level of tiny crack width was verified to be of little impact on the water permeability coefficient and chloride diffusion penetration.

Nevertheless, though ECC has such a high strain capacity, it still cannot be used as a structural material without steel reinforcement. UHPC does have the effect of enhancing the strength of structural members. But strength is not the only thing that is of concern in structural design. In most of cases, ductility is equally or even more important for structure. According to the ISO code [11], the strain capacity of steel used for reinforcing concrete structures (ductility class C) should be no less than 7% and 15% corresponding to the peak stress and at fracture. At present, the tensile strain capacities of the majority of existing fiber reinforced concrete (FRC), including ECC, are still far below this level. It implies that, when a structure made of FRC and steel reinforcement is loaded to its ultimate limit state, the steel reinforcement may sustain its contribution, but FRC will probably fail when subjected to excessive stretch. For the scenarios when longitudinal steel is not applicable for reinforcing concrete, for example, a structure worked in a high chloridion environment (coastal construction), concrete mixed with seawater and sea sand (isolated island construction), or 3D printing concrete, it is of great significance to find a cementitious material which has deformability matching that of steel, so as to make itself a structural material free from longitudinal reinforcement.

In previous studies, researchers and engineers used fly ash in high volumes (fly ash-to-cement ratio normally from 1.2 to 5.6) to produce ECC with excellent tensile ductility greenness [12,13]. The presence of spherical-shaped fly ash particles acting as smooth filler for the ECC matrix was verified effective in reducing the fracture toughness of the matrix and chemical bonding between polyvinyl alcohol (PVA) fiber and the surrounding matrix [14], and thus improving the ductility of ECC. However, the changes brought by fly ash incorporation may be not positive for another kind of ECC, i.e., polyethylene (PE) fiber reinforced ECC. It is well known that, for engineered cementitious composite reinforced with PVA fiber (PVA-ECC), the interfacial bond between fiber and matrix is excessive due to the hydrophilic nature of PVA. The excessive interfacial bond between fiber and matrix needs to be reduced so as to avoid premature rupture of fibers when fibers are pulled out from matrix [15]. In contrast, PE fiber has higher elastic modulus and tensile strength than PVA fiber. Hajiesmaeili and Denairé [16] studied the replacement of steel fibers by PE fibers to develop next generation strain hardening ultra high-performance fiber reinforced concrete (SH-UHPFRC) for sustainable structural applications. However, the hydrophobic nature of PE fiber induces weak bond at fiber/matrix interface [17,18,19]. In this case, the incorporation of fly ash may lead to negative effect to the tensile strain capacity of PE-ECC, therefore the concern turns to how to enhance the connection between fiber and matrix.

Furthermore, more than 2 billion tons of construction and demolition (C&D) waste are released in China every year. To date, most of the C&D waste are ended up by landfills, leading to water, atmospheric, and soil contamination [20]. For environmental protection concern, the C&D waste, particularly the fine particles with a size below 150 μm, which accounts for about 20% of the total amount, is badly needed to be reused [21]. However, despite their abundance in C&D waste, reuse of the waste particle (WP) are very limited in practical applications. In recent years, many investigators tried to use WP as supplementary cementitious material in producing concrete. Kwon and Schoon [22,23] studied the mechanical property of concrete incorporating WP and suggested that the replacement percentage of WP should be limited to a maximum value of 10%. Yong and Yun [24] indicated that WP obtained from waste mortar contains un-hydrated cement and thus can be recycled to replace up to 15% of the cement amount in concrete. Nevertheless, the replacement percentage of WP is still very low due to its inferior properties and larger particle size [25,26,27]. In attempt to solve this problem, the authors used ball grinding to reduce the particle size of WP to less than 45 μm, similar to the size of commercial fly ash but having much irregular morphology. In this article, the processed WP with the particle size less than 45 μm is referred as recycled powder (RP). More importantly, the RP with irregular morphology is expected to enhance the bond between PE fiber and matrix.

To improve the ductility of ECC which is already an order of magnitude higher than that of the normal FRC, some extra changes should be imposed to traditional ECC matrix. Crumb rubber (CR) incorporation is one of the choices. Annually, about 1.5 billion scrap tires were generated, in which the United States accounts for more than 300 million, and Europe accounts for more than 200 million. Generally, the way to dispose the scrap tires is by burning or burying, which causes pollution and is potentially hazardous to the environment [28,29]. Industries produce CR from scrap tires for reuse. Some researchers incorporated CR into ECC mixture to decrease the fracture toughness of the matrix, allow micro-cracks to be more easily triggered, thus favoring the multiple-cracking behavior of ECC, which in turn facilitates its deformability [30,31,32,33,34]. 

With all the above points in mind, the authors herein aim to use recycled powder and crumb rubber to substitute some of fly ash and silica sand in ECC mixture. The improved ECC is expected to have a tensile strain capacity up to 10%, which may match or even exceed the requirement for the deformability of steel reinforcement according to relative codes. By this way, ECC is expected of wider application range in civil engineering.

## 2. Materials and Experimental Methods

### 2.1. Materials and Mixture Proportions

**Figure 1:**
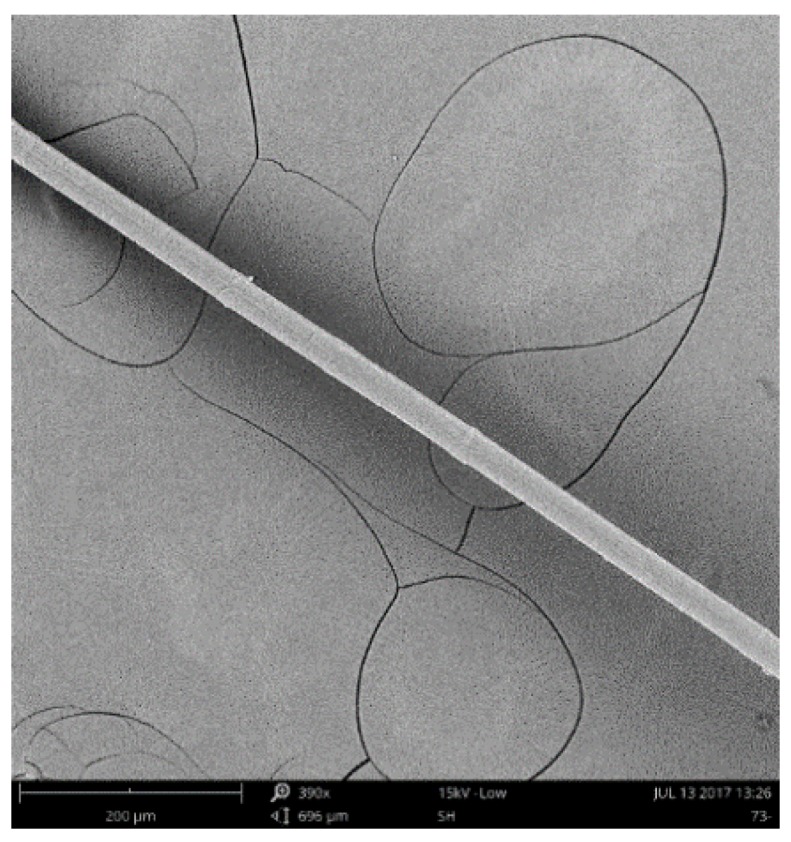
Longitudinal surface of PE fiber.

**Figure 2:**
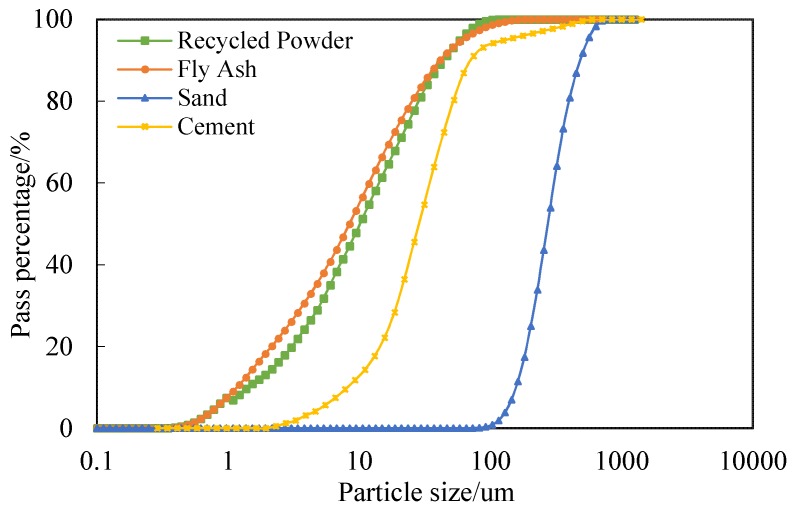
Particle size of ingredients.

**Figure 3:**
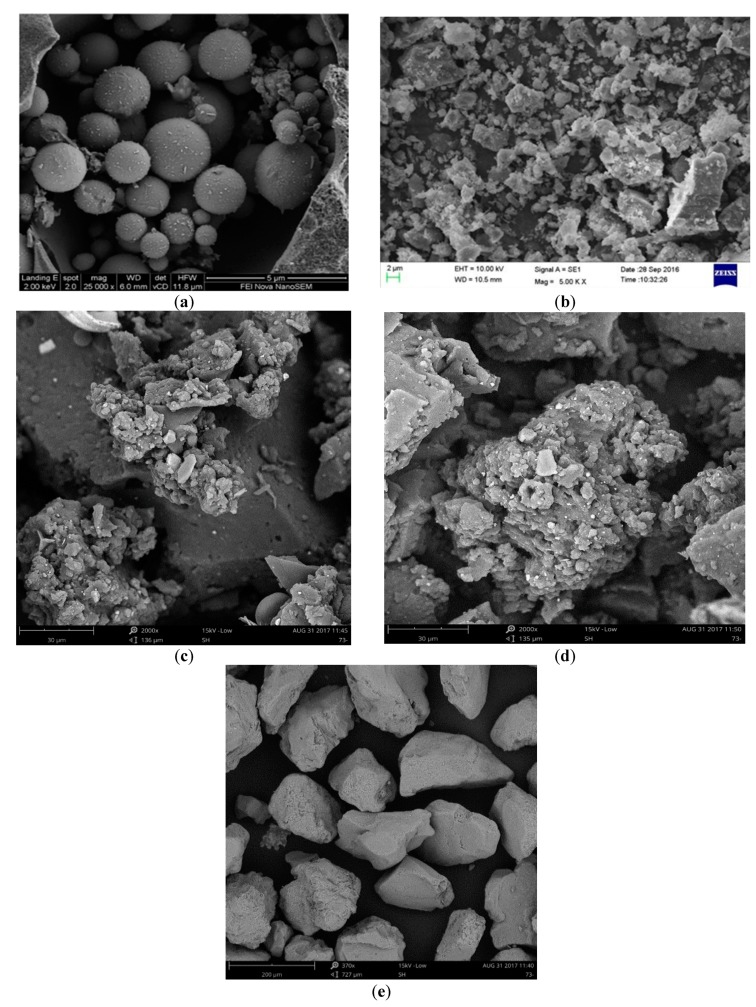
The morphologies of ingredients under SEM. (**a**) FA; (**b**) RP; (**c**) 40CR; (**d**) 80CR; (**e**) Sand.

The raw materials including Portland cement (PII. 52.5), class F fly ash (FA), recycled powder (RP), silica sand, crumb rubber (CR), water, polyethylene (PE) fiber, and polycarboxylic high-range water reducer (HRWR) were used in the production of ECC mixtures. Table 1 presents the geometric and mechanical properties of PE fiber used as reinforcement. According to the reference [35], using longer and thinner fibers helps to improve the tensile strain capacity and fracture energy of the composites. After extensive trial and error tests [36], this PE fiber type provided by Royal DSM (Heerlen, Netherlands) was used as reinforcement. In all the mixtures, the volume fraction of PE fibers was uniformly 2%. Figure 1 shows the PE fiber longitudinal image. The chemical compositions of the cement, FA and RP are listed in Table 2. The grain size distribution of aggregates and cementitious materials are presented in Figure 2. The size of two kinds of CR are 40 mesh and 80 mesh, respectively. The particle size of 80CR is smaller than 40CR. The morphologies of FA, RP, silica sand, and CR observed under the scanning electron microscope (SEM, Phenom ProX, Phenom-World, Eindhoven, Netherlands) were seen in Figure 3, which illustrate that the processed RP and CR have extremely irregular microstructure, as compared with the spherical-shaped FA and silica sand. 

In accordance with the ECC design theory [37], the macroscopic tensile strain hardening behavior of ECC is strongly influenced by the characteristics of fiber, matrix and fiber/matrix interface. In this study, matrix and interface properties were tailored through adjusting the RP replacement percentage (50%, 100% by weight of the fly ash), as well as the CR replacement ratio (13%, 30% by weight of the silica sand) and size (80 mesh and 40 mesh). In total, the test matrix includes eight ECC mixtures as listed in Table 3. Each mixture is named as the corresponding symbol in terms of its composition characteristics, for example, R_50_80C_13_ represents 50% RP replacement percentage, 80 mesh CR and 13% CR replacement ratio. 

### 2.2. Specimen Preparation

All ECC mixtures were prepared using a Hobart mixer with 20-liter capacity. Firstly, the solid ingredients including the cement, fly ash, recycled powder, silica sand, and CR were mixed for 2 min. Then water and HRWR was added and mixed into the mixture until the fresh paste was homogenous and consistent. Finally, PE fibers were gradually added into the mortar and mixed for 3 minutes to ensure good dispersion. Afterward, the mixture was cast into steel molds and covered with plastic sheets. All the specimens were de-molded after 1 day, and then cured in air at room temperature of 20 ± 3 °C and relative humidity (RH) of 40 ± 5% until the prescribed testing age of 28 days.

### 2.3. Testing Procedures

Experimental investigations, including uniaxial tensile, compressive test, single crack tension test, and matrix fracture toughness test were carried out in present research. Due to the variable of different ECC materials, at least four specimens are prepared for each case.

**Figure 4:**
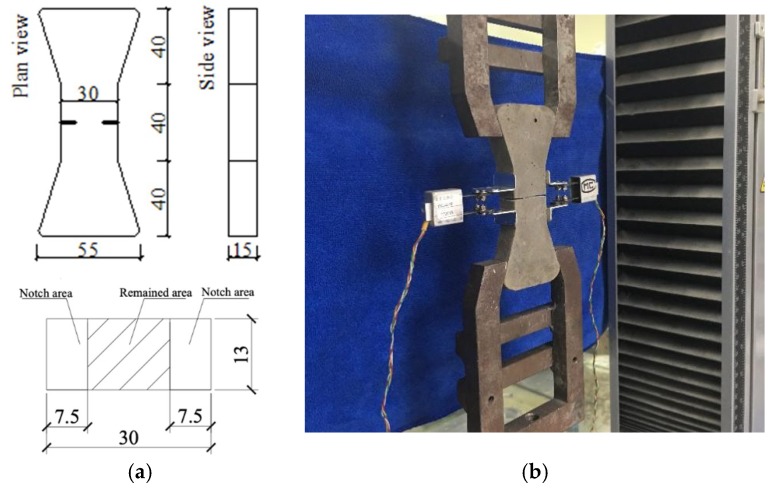
Single-crack specimen and tension test of ECC. (**a**) Geometry of notched dogbone specimen; (**b**) test setup. (unit: mm).

In the theory of ECC design, the fiber-bridging relationship determines the value of the fiber bridging complementary energy *J*_b_’, which is of primary importance. It describes the relationship between the bridging stress σ transferred across a crack and the opening of this crack *δ*. To improve the ductility of conventional ECC using RP and CR, it is necessary to experimentally determine the relevant σ-δ curve. Therefore, single-crack tension tests were conducted to investigate the collective bridging behavior of multiple fibers across a crack in different ECC mixtures. The single crack tension test is to control the notched specimen to generate only one single crack in uniaxial tension. Minor dog-bone specimens (fiber volume fraction of 1%) were used and notched on two lateral faces to deliberately enforce a single crack. Figure 4 shows the specimen geometry and test setup schematically. An ultra-thin saw blade (0.4 mm in thickness) was used to cut notch with a width less than 0.6 mm. To avoid extra crack occurring inside or outside the notch, the adopted dimensions allowed the reduction of cross-sectional area to 50% of its initial value. Ideally, a single crack should occur at notch when the uniaxial tensile load is applied. Two clip-on gages with a uniform gauge length of 5 mm measures the crack opening displacement in this kind of test.

**Figure 5:**
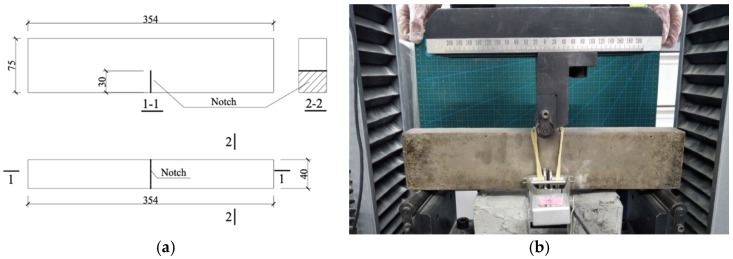
The setup of three-point bending test. (**a**) Geometry of notched beam; (**b**) test setup. (unit: mm).

To figure out the influence of RP and CR on matrix toughness with different mixtures in this paper, three-point bending tests were conducted to obtain the matrix fracture energy *J*_tip_ and the matrix fracture toughness *K*_m_ on notched beams, following to ASTM E399 [38].The fracture toughness beams (without fiber) measure 354 × 75 × 40 mm^3^, with a span of 320 mm between supports. Before testing, an electrical diamond saw was used to cut a 30.0 mm depth notch into the middle bottom of specimen. One clip-on gage was installed to the lateral surface to measure the crack opening displacement. The test setup and the specimen size are shown in Figure 5.

**Figure 6:**
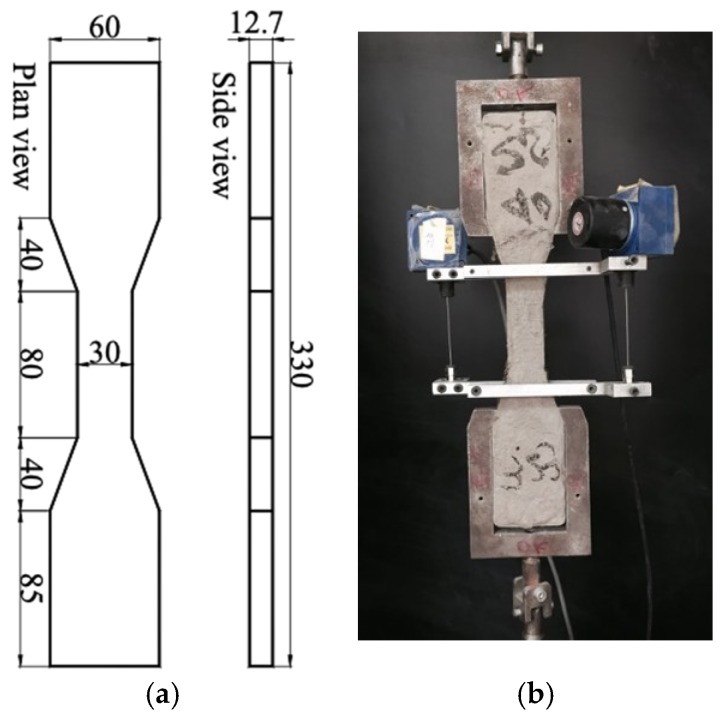
Dogbone specimen for ECC tensile test. (**a**) Dimension of dogbone specimen; (**b**) test setup. (unit: mm).

According to the method recommended by the Japan Society of Civil Engineers [39], dog-bone shaped specimen was tested in uniaxial tension as shown in Figure 6. A MTS CMT4204 electro-servo machine (±1 N resolution) was employed to apply displacement-control at the rate of 0.5 mm/min. Two wired electronic coders, fixed on the lateral faces with a gauge length of 80 mm, are used to measure the deformation of the specimen during test process.

**Figure 7:**
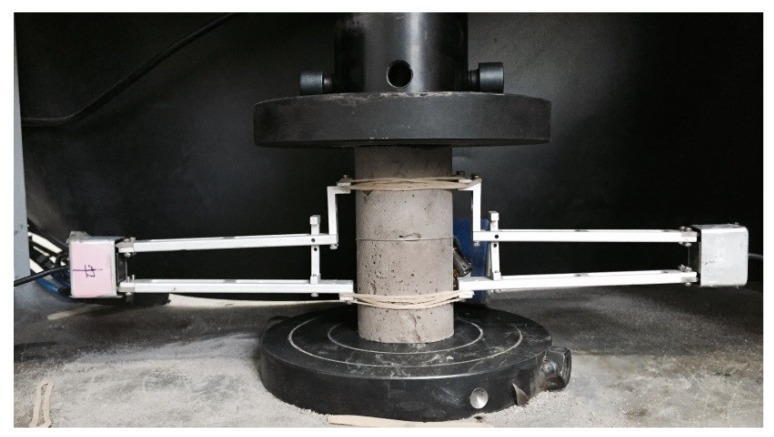
Compressive test of ECC.

For each mixture, four cylinders with a size of ϕ 50 mm × 100 mm were tested to obtain the compressive performance using a MTS 244 electro-hydraulic servo machine (±100 N resolution). The compression speed was 2 mm/min. Two clip-on gages with a gauge length of 60 mm were installed to the lateral surface to measure the vertical deformation of cylinder in compression. The compressive test set-up is presented in Figure 7.

## 3. Results and Discussion

### 3.1. Composites Tensile Properties

**Figure 8:**
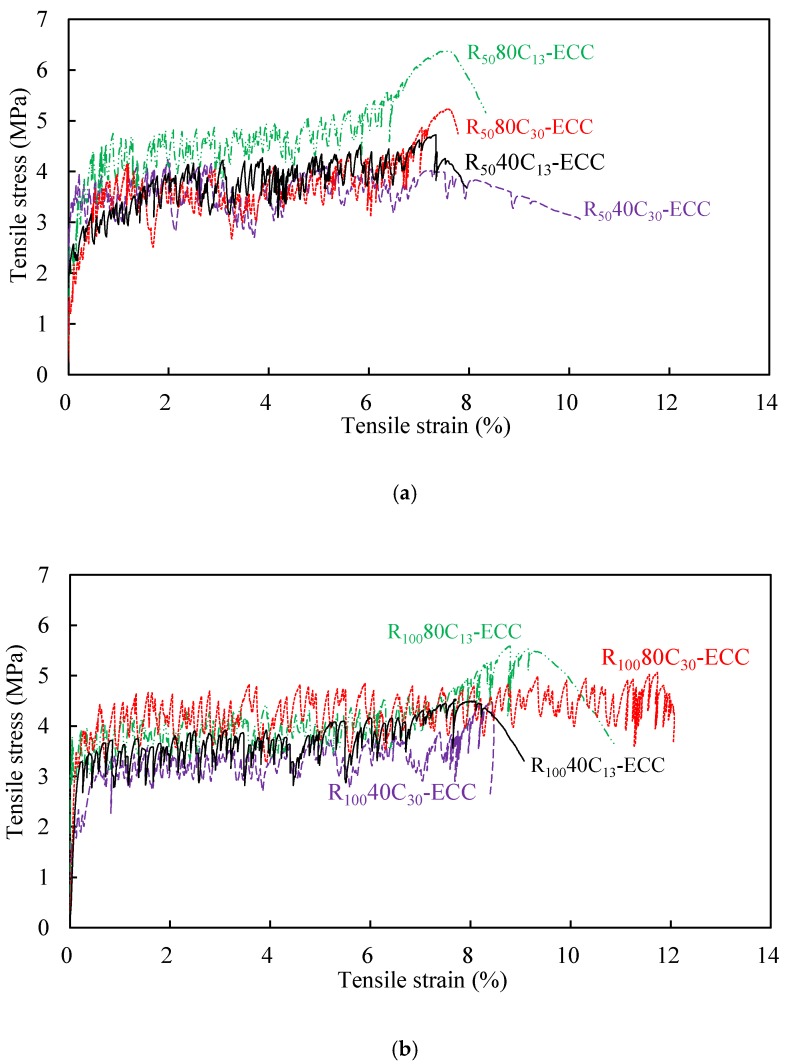
Tensile stress–strain curves of all the mixtures. (**a**) Tensile behavior of ECC with 50% RP replacement ratio; (**b**) tensile behavior of ECC with 100% RP replacement ratio.

Figure 8 shows the typical tensile stress–strain curves of all the mixtures obtained from uniaxial tension test. It is seen that all mixtures exhibit significant strain-hardening behavior and high ductility with multiple cracks, which is unlike the sudden brittle fracture failure mode for normal concrete. The strain capacity of the mixtures observed in Figure 8 is obviously higher than 3%, which is normally reported for PVA-ECC in the previous literature [14]. Three distinct phases can be observed within each sample, which are the elastic stage, the strain-hardening stage, and the failure stage. During the strain-hardening stage, the tensile stress has the larger fluctuation than that observed in PVA-ECC [30]. This phenomenon is due to the hydrophobic nature of PE fiber used in this study, which leads to a much weaker bond between fiber and matrix, as compared with that from PVA fiber [36]. However, there is a high chemical bond between PVA fiber and the matrix [13,40]. Therefore, it is generally the case that there is a relatively high stress drop when a crack occurs in a composite reinforced by PE fiber.

The tensile test results in terms of initial cracking stress *σ*_tc_, peak stress *σ*_tu_, tensile strain capacity *ε*_tu_ (e.g., strain at peak stress), crack number *N*_c_, crack width *w*_c_ and crack spacing *s*_c_ are summarized in Table 4. By counting crack numbers within 80 mm gauge length after tensile tests, the average crack width and crack spacing were calculated. As can be seen in Table 4, the crack widths of all mixtures were less than 150 μm at peak tensile strength, indicating the excellent crack width control of the developed ECC. The strain capacity of ECC is dependent on the density of multiple cracking and crack width distribution. R_50_-ECC and R_100_-ECC had the similar crack width, but R100-ECC showed more crack numbers, demonstrating that the increase of RP content not only did not impair on the multi-cracking characteristics of ECC, but it triggered more cracks instead. The tensile strain capacity of all the composites ranged from 7% to 12%, at least ten times higher than that of normal fiber reinforced concrete. Compared with the ECC without RP and CR whose tensile strain capacities are approximately 3–5% [14,41], the developed ECC shows significant advantage in tensile ductility. These indicated that the addition of RP and CR did do a positive effect on the improvement of normal ECC’s ductility. 

The value of initial cracking stress was determined from the beginning point of the strain hardening branch of the stress–strain curves. It is clearly observed that all the values of the peak tensile stress were about 2–3 times higher than the initial cracking stress, indicating that all the composites satisfied the strength criterion of multiple cracking [42]. As a result, tensile strain capacity of all the composites could reach over 7%.

**Figure 9:**
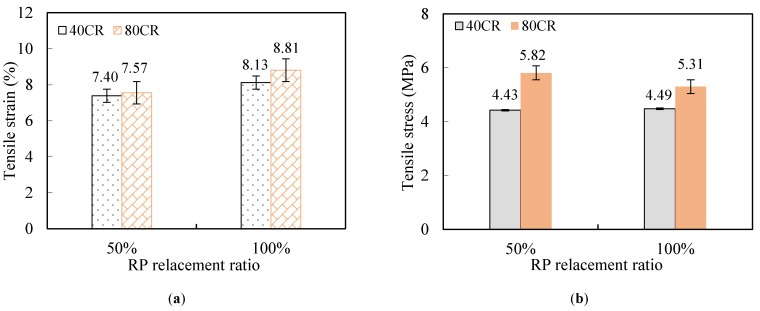

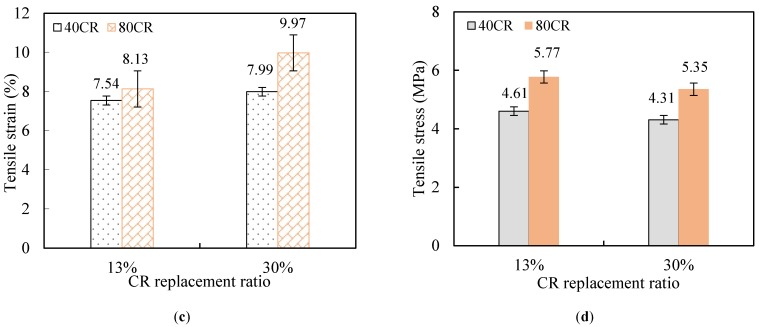
Tensile property of ECC with different RP and CR content. (**a**) Tensile strain versus RP replacement ratio; (**b**) tensile stress versus CR replacement ratio; (**c**) tensile strain versus CR replacement ratio; (**d**) tensile stress versus CR replacement ratio.

Figure 9 shows the effect of different RP replacement ratio, CR content and CR size on the tensile peak stress and strain capacity of ECC. When fly ash was partially or totally replaced by RP, the strain capacity is significantly improved as increasing the content of RP, denoting that the irregular microstructure of RP enhanced the interface bonding between fiber and matrix, see Figure 3. Similarly, the strain capacity has a marked increase when the CR content increases, owing to the fact that using rubber powder replacing sand lowers the toughness of matrix. When it comes to the CR particle size, it could be found that the strain capacity is more pronounced when the smaller particle size (80CR) is used. A possible explanation is that finer CR particles entrain more air bubble into ECC since the air bubble is likely to be attached on the surface of CR particle during mixing process [31], which increase the number of small flaws in ECC matrix. And these would make it easier to initiate crack, leading to benefit the ductility of ECC. The improvement of ductility with the addition of RP and CR can be explained from the micro-mechanics design theory of ECC materials. And a further discussion is presented in Section 3.3. However, the increase of RP or CR content has no positive effect on the initial cracking stress and peak stress, even a slight decrease.

### 3.2. Composite Compressive Property 

**Figure 10:**
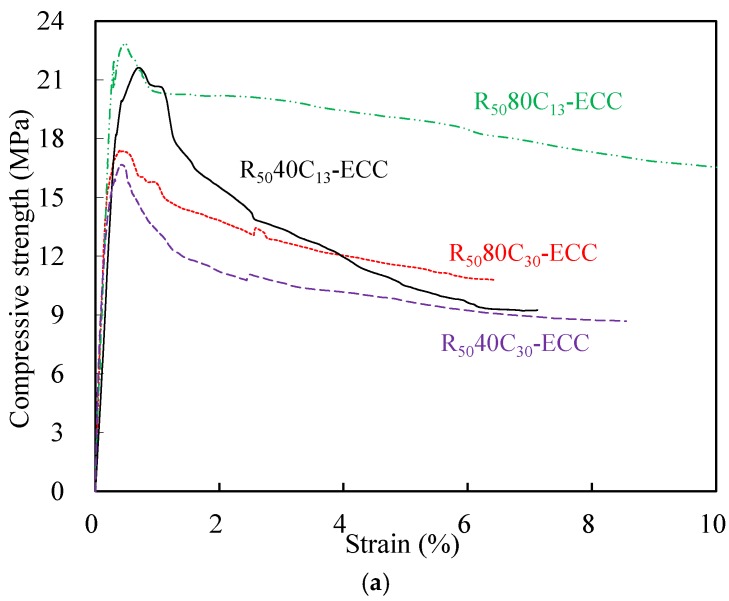

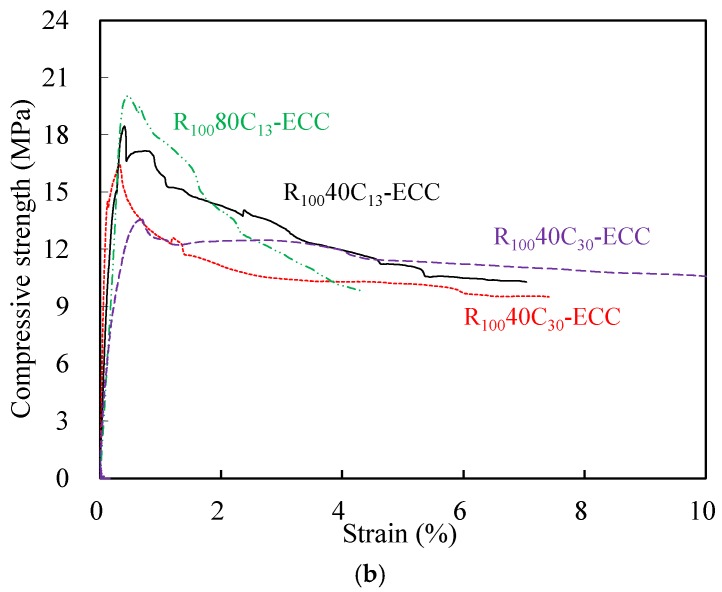
Compressive strain–stress curves of ECC cylinders. (**a**) Compressive behavior of ECC with 50% RP replacement ratio; (**b**) compressive behavior of ECC with 100% RP replacement ratio.

The compressive stress–strain curves of different mixtures are illustrated in Figure 10. As can be seen, the behaviors of the ECC prior to the peak stress was similar to that of the normal concrete, but the post-peak softening behaviors are quite different. Comparatively, the descending rates of all the mixtures were much gentler, exhibiting their excellent post-peak deformation capacity under compression. According to the data from the compressive stress–strain curves, the strain at peak compressive stress of R_50_80C_13_-ECC and R_100_40C_30_-ECC were 0.72% and 0.65% and the strain at 80% peak compressive stress of R_50_80C_13_-ECC and R_100_40C_30_-ECC were 6.15% and 4.06%, respectively, about one order of magnitude higher than that of normal concrete. The improvement of compressive deformability of composites can be mainly attributed to the superior crack bridging capacity provided by PE fibers as well as the addition of CR. 

**Figure 11:**
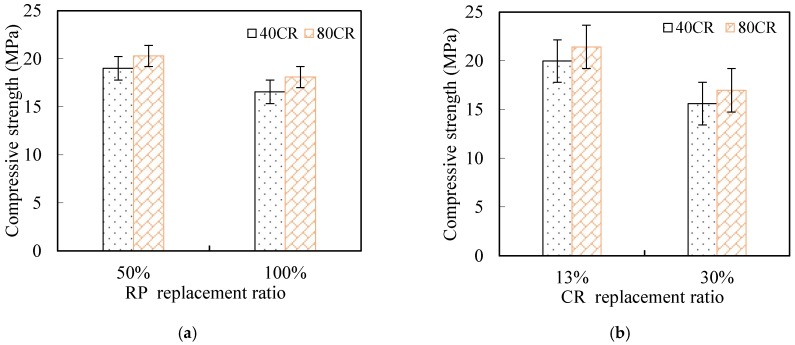
Compressive property of ECC with different RP and CR content. (**a**) Compressive property vs. RP replacement ratio; (**b**) compressive property vs. CR replacement ratio.

The influence of replacement ratios and CR sizes on the compressive properties of the developed ECC are illustrated in Figure 11. As shown in Figure 11, with the RP replacement ratio rising from 50% to 100%, the compressive strength of composites incorporating 40CR and 80CR dipped by 21.9% and 20.8%, respectively. Though the considerable SiO_2_ contents in RP, which possess filling and pozzolanic effects, can improve the concrete mechanical properties, the positive effects of RP were taken over by the excessive contents of inactive CaCO_3_ when the RP replacement percentage reached 100%. Therefore, identifying the optimal percentage of the RP for a particular mix is of great importance. Similarly, there was a slight decline in the compressive strength of ECC mixtures with the increase of CR replacement ratio. The decrease in compressive strength is due to the increase of porosity caused by the addition of crumb rubber. In terms of the CR particle size, it could be found that the higher compressive strength can be achieved when the finer CR particle size (80CR) partially replaced sand.

It is noted that the ratios of the tensile strength to the compressive strength of the composites ranged from 21% to 32%, which are obviously higher than the usual ratio (about 10%) of normal concrete. This is due to the tensile strength of ECC is mainly dependent on the fiber bridging capacity rather than matrix strength. It is observed that the compressive strain at peak strength is significantly enhanced with the increase of CR content, especially when smaller size CR was used. And these should be due to the much lower elastic modulus and better deformation capacity of CR than silica sand.

### 3.3. Fiber Bridging Capacity and Fracture Toughness

Unlike the typical trial-and-error material development methodology, ECC is designed based on micromechanics, which links the microstructure at the micro scale with the composite behavior at the macro scale. The tensile strain hardening behavior of ECC can be achieved by satisfying two fundamental criteria, that is, strength criterion and energy criterion [42]. The strength criterion requires the fiber bridging strength to be higher than the matrix cracking strength, and all the composites proposed in this paper has been shown to meet this condition in Section 3.1. The energy criterion requires the fiber bridging complementary energy *J*_b_’ to be far greater than the matrix fracture energy *J*_tip_ to form steady state crack mode. Therefore, the ductility improvement in ECC incorporating RP and CR may be explained by using the two fundamental criteria and the classical PSH index [43].

**Figure 12:**
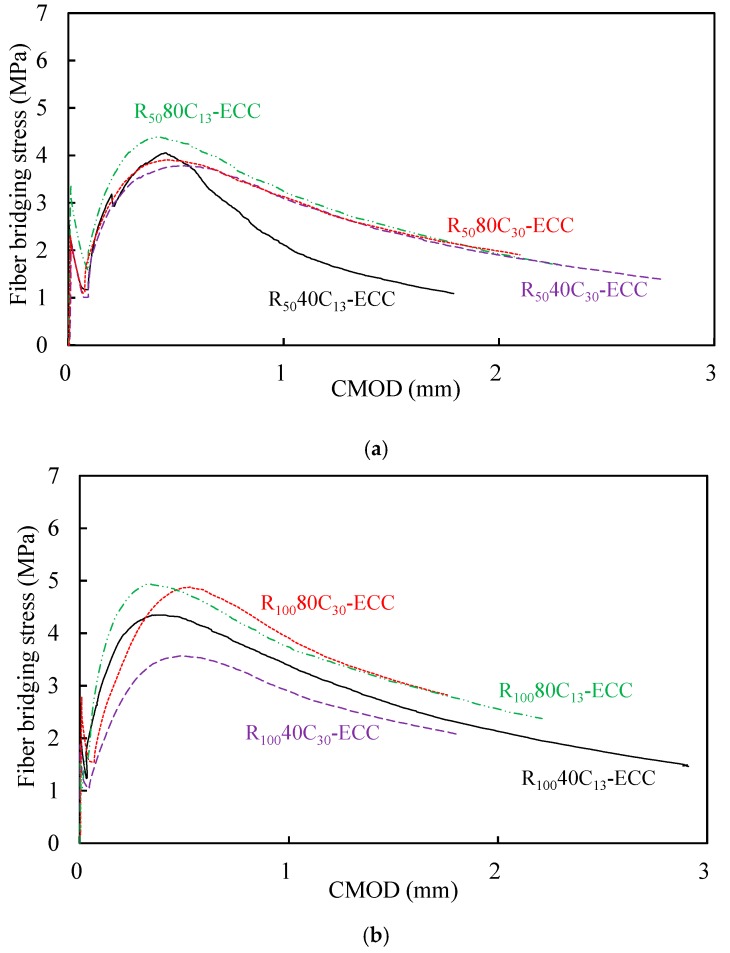
Single-crack tension test. (**a**) Fiber bridging behavior of ECC with 50% RP replacement ratio; (**b**) Fiber bridging behavior of ECC with 100% RP replacement ratio.

The fiber bridging stress *σ*_OC_ versus crack mouth opening displacement (CMOD) *δ*_B_ relation curves illustrated in Figure 12 were experimentally determined from single-crack tensile test. According to Equation (1), *J*_b_*’,* which is the reflection of energy consumed by friction when fibers are pulled out from matrix, can be derived.

(1) $$J_{tip}\leq J_{b}^{\prime }=\sigma_{B,\max}\delta_{B}-\int_{0}^{\delta_{B}}\sigma_{B}(\delta )d\delta$$

Table 5 lists the values of the peak stress *σ*_OC_, the corresponding crack opening displacement *δ*_B_ and *J*_b_’. As shown in Table 5, it is clear that the values of *J*_b_’ are obviously enhanced with the increasing replacement ratio of RP, which demonstrated that RP does have effect of increasing the bond-slip capacity between fiber and matrix, especially in the case that the smooth and spherical-shaped fly ash was replaced by the irregular-shaped RP particles, see Figure 3. However, there was a slight decrease of fiber bridging stress *σ*_OC_ when the CR replacement ratio changed from 13% to 30%, indicating that excessive contents of CR would generate negative effect on the interfacial bond between PE fiber and matrix. Despite the reduction of fiber bridging peak stress *σ*_OC_ as the increase of CR replacement ratio, the value of *J*_b_’ still had a slight improvement due to the remarkable rise of the *δ*_B_ value.

Referring to the three-point bending test in ASTM E399 [38], the matrix fracture toughness K_Q_ can be derived according to Equations (2) and (3).

(2) $$K_{Q}=\frac{P_{Q}S}{\sqrt{BB_{N}}W^{3/2}}\cdot f\left(\frac{a}{W}\right)$$

(3) $$f\left(\frac{a}{W}\right)=3\sqrt{\frac{a}{W}}\cdot \frac{1.99-\left(\frac{a}{W}\right)\left(1-\frac{a}{W}\right)\left[2.15-3.93\frac{a}{W}+2.7{\left(\frac{a}{W}\right)}^{2}\right]}{2\left(1+2\frac{a}{W}\right){\left(1-\frac{a}{W}\right)}^{3/2}}$$

where *P*_Q_ is the peak load; *S* is the span of the three-point beam; *a* is the notch depth; *W* is the width of the three-point beam; *B* and *B*_N_ are the thickness of the three-point beam; *f*(*α/W*) is the shape parameter of three-point beam. 

Based on the maximum load *K*_Q_, the matrix fracture energy *J*_tip_ was computed according to Equation (4), where *E*_m_ is the elastic modulus of matrix determined from the initial slope of the tensile stress–strain curve.

*J*_tip_ = *K_Q_*^2^/*E*_m_ (4)

The related calculation parameters of the notched beam specimen and the fracture indexes (*K*_Q_ and *J*_tip_) are given in Table 6. As can be seen, the fracture indexes had a modest decline as the increasing of RP replacement ratio. It can be explained that RP has the close content of SiO_2_, but the higher content of CaCO_3_ (CaO) in comparison with the fly ash presented in Table 2. As can be seen, the fracture indexes had a modest decline with the increase of the RP replacement ratio. It can be explained that RP has the close content of SiO_2_, but the higher content of CaCO_3_ (CaO) in comparison with the fly ash presented in Table 2. Similarly, the addition of CR diminished the matrix toughness *K*_Q_ and the matrix fracture energy *J*_tip_, which should attribute to the low elastic modulus of CR and weak bonging between CR particle and matrix. To a certain extent, the CR powder can be considered as the artificial flaw and it can make matrix easier to crack.

**Figure 13:**
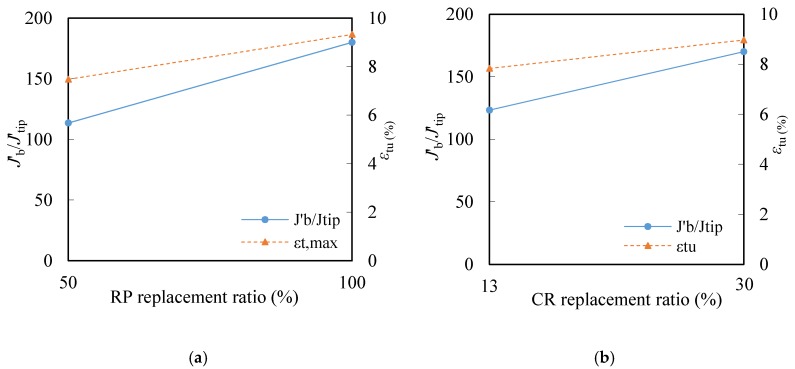
Relations between *J*_b_’/*J*_tip_ and *ε*_tu_. (**a**) RP replacement ratio; (**b**) CR replacement ratio.

According to the pseudo-strain hardening (PSH) criterion proposed by Kanda and Li [43], the tensile strain capacity of cementitious composites is closely related to the ratio *J*_b_’/*J*_tip_ (PSH index), which indicates the potential of developing multiple cracking. Specifically, a higher *J*_b_’/*J*_tip_ ratio is favorable for saturated multiple cracking behavior and leads to higher strain capacity. *J*_b_’, *J*_tip_, tensile strain capacity *ε*_tu_, and the values of *J*_b_’/*J*_tip_ are listed in Table 7. Figure 13 shows the relation between the tensile strain capacity *ε*_tu_ and the strain hardening index *J*_b_’/*J*_tip_. It is observed that *J*_b’_/*J*_tip_ remains the positive correlation with tensile capacity. The higher *J*_b_’/*J*_tip_ ratio unsurprisingly leads to higher strain capacity. The ratio of *J*_b’_/*J*_tip_/*ε*_tu_ ranges from 12.4 to 23.8, demonstrating the PSH index still acceptable as a criterion to quantify the tensile ductility of composites.

In summary, the incorporation of RP increases *J*_b_′ and the incorporation of CR decreases *J*_tip_ result in enhanced strain-hardening PSH index. Therefore, combining effect of decreasing *J*_tip_ and increasing *J*_b_’ leads to a larger *J*_b_’/*J*_tip_, indicating a better chance for saturated multiple cracking and significant improvement ductility.

## 4. Conclusions

In this paper, the feasibility of using recycled powder and crumb powder to improve the greenness of ECC and enhance the deformation capacity of ECC was verified. A series of experiments were conducted to investigate the influences of RP and CR on the mechanical properties of ECC. The specific conclusions can be drawn as following:

(1) ECC incorporating recycled powder and crumb rubber exhibited strain hardening behavior and saturated crack pattern. The crack widths of all the composites were all less than 150 μm.
(2) The deformation capacity is improved as increasing the content of recycled powder. To some degree, the addition of the irregular-shaped RP particles tends to increase the fiber bridging complementary energy and trigger more cracks.
(3) Incorporating crumb rubber increased the strain capacity, while sharply decreased the matrix toughness *K*_Q_ and the matrix fracture energy *J*_tip_. And the effect of crumb rubber on the tensile strain capacity is more pronounced when the smaller particle size is used. To a certain extent, the crumb rubber powder can be considered as the artificial flaw and it can make matrix easier to crack.
(4) Due to the use of recycled powder and crumb rubber, ECC is expected to be a more environmentally friendly material.

## Figures and Tables

**Table 1:** Properties of polyethylene (PE) fiber.

Length (mm)	Diameter (μm)	Fiber Aspect Ratio	Nominal Strength (GPa)	Modulus (GPa)	Rupture Elongation (%)	Density (g/cm^3^)
18	25	720	2.9	116	2.42	0.97

**Table 2:** Chemical compositions of raw materials (wt %).

Ingredients	Na_2_O	MgO	Al_2_O_3_	SiO_2_	P_2_O_5_	SO_3_	K_2_O	CaO	TiO_2_	MnO	Fe_2_O_3_
**Cement**	0.08	0.66	4.42	19.9	0.1	2.67	0.79	64.9	0.21	0.1	3
**Fly ash**	0.58	0.9	23.9	51.7	0.4	0.91	1.40	7.65	1.19	0.07	5.22
**Recycled powder**	0.86	2.26	12	47.9	0.29	1.41	2.33	18.7	0.82	0.1	6.53

**Table 3:** Mixture design. CR: crumb rubber, ECC: engineered cementitious composites.

Mixture ID	Binder			80CR	40CR	Sand	Water	PE Fibers
	Cement	Fly ash	Recycled Powder					
R_50_40C_13_-ECC	0.45	0.275	0.275	-	0.04	0.27	0.35	0.015
R_50_40C_30_-ECC	0.45	0.275	0.275	-	0.08	0.23	0.37	0.015
R_50_80C_13_-ECC	0.45	0.275	0.275	0.04	-	0.27	0.40	0.015
R_50_80C_30_-ECC	0.45	0.275	0.275	0.08	-	0.23	0.36	0.015
R_100_40C_13_-ECC	0.45	-	0.55	-	0.04	0.27	0.34	0.015
R_100_40C_30_-ECC	0.45	-	0.55	-	0.08	0.23	0.37	0.015
R_100_80C_13_-ECC	0.45	-	0.55	0.04	-	0.27	0.39	0.015
R_100_80C_30_-ECC	0.45	-	0.55	0.08	-	0.23	0.34	0.015

All numbers are mass of binder weight.

**Table 4:** Results of uniaxial tension test (average value).

Mixture ID	*σ*_tc_ (MPa)	*σ*_tu_ (MPa)	*ε*_tu_ (%)	*N*_c_	*w*_c_ (μm)	*s*_c_ (mm)
R_50_40C_13_-ECC	2.22	4.62	7.37	61	96.7	1.96
R_50_40C_30_-ECC	1.97	4.24	7.42	66	89.9	1.96
R_50_80C_13_-ECC	2.12	6.00	7.45	64	93.1	1.82
R_50_80C_30_-ECC	1.72	5.63	7.68	62	99.1	1.94
R_100_40C_13_-ECC	2.23	4.59	7.71	67	92.1	1.72
R_100_40C_30_-ECC	1.72	4.38	8.55	70	97.7	1.95
R_100_80C_13_-ECC	1.96	5.54	8.81	72	97.9	1.72
R_100_80C_30_-ECC	2.21	5.07	12.26	78	125.7	1.83

**Table 5:** Test results of single-crack tension test.

Mixture ID	*σ*_OC_ (MPa)		*δ*_B_ (mm)		*J_b_’* (J/m^2^)
	Average	Variance	Average	Variance	
R_50_40C_13_-ECC	4.01	0.898	0.45	0.097	1208.7
R_50_40C_30_-ECC	3.76	0.454	0.55	0.062	1392.7
R_50_80C_13_-ECC	4.20	0.42	0.41	0.126	1055.8
R_50_80C_30_-ECC	3.96	0.808	0.50	0.115	1182.0
R_100_40C_13_-ECC	4.35	0.358	0.39	0.021	1223.4
R_100_40C_30_-ECC	3.89	0.528	0.52	0.095	1405.5
R_100_80C_13_-ECC	4.94	0.133	0.33	0.051	1275.8
R_100_80C_30_-ECC	4.88	0.014	0.55	0.408	1429.2

**Table 6:** Fracture toughness of mixtures (in average value).

Mixture ID	*α* (mm)	*W* (mm)	*S* (mm)	*P*_Q_ (N)	*K*_Q_ (MPa·m^1/2^)	*J*_tip_ (J/m^2^)
R_50_40C_13_-ECC	30	75	320	469	0.362	13.1
R_50_40C_30_-ECC	30	75	320	463	0.357	12.7
R_50_80C_13_-ECC	30	75	320	438	0.338	11.4
R_50_80C_30_-ECC	30	75	320	360	0.272	7.4
R_100_40C_13_-ECC	30	75	320	280	0.287	7.7
R_100_40C_30_-ECC	30	75	320	340	0.263	6.9
R_100_80C_13_-ECC	30	75	320	378	0.292	8.5
R_100_80C_30_-ECC	30	75	320	340	0.262	6.9

**Table 7:** Comparisons on pseudo-strain hardening (PSH) index.

Mixture ID	*J*_b_’ (J/m^2^)	*J*_tip_ (J/m^2^)	*J*_b_ *’/J*_tip_	*ε*_tu_ (%)	*J*_b_*’/J*_tip_/*ε*_tu_
R_50_40C_13_-ECC	1208.7	13.1	92.3	7.37	12.52
R_50_40C_30_-ECC	1392.7	12.7	109.7	7.42	14.78
R_50_80C_13_-ECC	1055.8	11.4	92.6	7.45	12.43
R_50_80C_30_-ECC	1182.0	7.4	159.7	7.68	20.80
R_100_40C_13_-ECC	1223.4	7.7	158.9	7.71	20.61
R_100_40C_30_-ECC	1405.5	6.9	203.7	8.55	23.82
R_100_80C_13_-ECC	1275.8	8.5	150.1	8.81	17.04
R_100_80C_30_-ECC	1429.2	6.9	207.1	12.26	16.89
